# Analyzing Reddit Forums Specific to Abortion That Yield Diverse Dialogues Pertaining to Medical Information Seeking and Personal Worldviews: Data Mining and Natural Language Processing Comparative Study

**DOI:** 10.2196/47408

**Published:** 2024-02-14

**Authors:** Danny Valdez, Lucrecia Mena-Meléndez, Brandon L Crawford, Kristen N Jozkowski

**Affiliations:** 1 Department of Applied Health Science Indiana University School of Public Health Bloomington, IN United States

**Keywords:** abortion, social media, Reddit, natural language processing, NLP, neural networks

## Abstract

**Background:**

Attitudes toward abortion have historically been characterized via dichotomized labels, yet research suggests that these labels do not appropriately encapsulate beliefs on abortion. Rather, contexts, circumstances, and lived experiences often shape views on abortion into more nuanced and complex perspectives. Qualitative data have also been shown to underpin belief systems regarding abortion. Social media, as a form of qualitative data, could reveal how attitudes toward abortion are communicated publicly in web-based spaces. Furthermore, in some cases, social media can also be leveraged to seek health information.

**Objective:**

This study applies natural language processing and social media mining to analyze Reddit (Reddit, Inc) forums specific to abortion, including r/Abortion (the largest subreddit about abortion) and r/AbortionDebate (a subreddit designed to discuss and debate worldviews on abortion). Our analytical pipeline intends to identify potential themes within the data and the affect from each post.

**Methods:**

We applied a neural network–based topic modeling pipeline (BERTopic) to uncover themes in the r/Abortion (n=2151) and r/AbortionDebate (n=2815) subreddits. After deriving the optimal number of topics per subreddit using an iterative coherence score calculation, we performed a sentiment analysis using the Valence Aware Dictionary and Sentiment Reasoner to assess positive, neutral, and negative affect and an emotion analysis using the Text2Emotion lexicon to identify potential emotionality per post. Differences in affect and emotion by subreddit were compared.

**Results:**

The iterative coherence score calculation revealed 10 topics for both r/Abortion (coherence=0.42) and r/AbortionDebate (coherence=0.35). Topics in the r/Abortion subreddit primarily centered on information sharing or offering a source of social support; in contrast, topics in the r/AbortionDebate subreddit centered on contextualizing shifting or evolving views on abortion across various ethical, moral, and legal domains. The average compound Valence Aware Dictionary and Sentiment Reasoner scores for the r/Abortion and r/AbortionDebate subreddits were 0.01 (SD 0.44) and −0.06 (SD 0.41), respectively. Emotionality scores were consistent across the r/Abortion and r/AbortionDebate subreddits; however, r/Abortion had a marginally higher average *fear* score of 0.36 (SD 0.39).

**Conclusions:**

Our findings suggest that people posting on abortion forums on Reddit are willing to share their beliefs, which manifested in diverse ways, such as sharing abortion stories including how their worldview changed, which critiques the value of dichotomized abortion identity labels, and information seeking. Notably, the style of discourse varied significantly by subreddit. r/Abortion was principally leveraged as an information and outreach source; r/AbortionDebate largely centered on debating across various legal, ethical, and moral abortion domains. Collectively, our findings suggest that abortion remains an opaque yet politically charged issue for people and that social media can be leveraged to understand views and circumstances surrounding abortion.

## Introduction

### Background

Although the abortion debate is often framed along strict proabortion or antiabortion stances (eg, prochoice versus prolife—terms common in the United States, Ireland, and other English-speaking countries; *pro-elección* versus *provida* and *pro-aborto* versus *anti-aborto*—terms used in Mexico, Argentina, and other Spanish-speaking countries), actual abortion beliefs are complex, contextual, and at times contradictory [1-4]. Notably, despite media characterizations of these 2 oppositional perspectives—for people ascribing to either proabortion or prochoice labels (ie, broad abortion support) or antiabortion or prolife labels (ie, broad abortion opposition)—there exist circumstances in which people’s views diverge from the dichotomy [5]. These circumstances include, for example, the gestation period of pregnancy [6], the context for seeking abortion [7], and whether people consider abortion as a legal versus moral issue [8]. In addition, attitudes toward abortion also vary across some demographic characteristics such as age, educational attainment, political affiliation, and race or ethnicity of a person or groups of people participating in a survey [1,9,10].

Beyond context-specific or cultural considerations that may predict complex abortion views, personal accounts, narratives, and discussions about abortion may similarly reveal the extent to which abortion views depart from a support or opposition dichotomy, including extreme abortion circumstances or personal experience with an abortion. Evidence suggests that these considerations are not ethnocentric but shared globally. Research comparing abortion beliefs between English-speaking US residents and Spanish-speaking US residents of diverse nations of origin demonstrates that clear general differences exist in abortion beliefs. Following investigations of the abovementioned considerations, we suggest that further research may yield more precise insights into evolving views on abortion [11].

Contextual, contradictory, and, in some cases, changing beliefs on abortion make it difficult to accurately assess global and US abortion climates beyond rote and dichotomized categories [12]. However, evidence strongly suggests the US and global populations hold views that depart from these 2 categories, reflecting abortion attitude complexity [1,10]. Although survey data have quantitatively supported the idea of abortion attitude complexity—qualitative data, broadly defined as any type of open-ended text, audio, visual, or language data, may add additional nuance to suggest where and how complexity may emerge. For example, interviews about abortion reveal specific circumstances that contribute to variability in people’s views on abortion [4] or reveal how current events and news cycles, in turn, shape social beliefs and attitudes [13]. Qualitative data can also inform how people contextualize assistance-related resources such as those found on social media.

Social media posts, as a novel form of qualitative data, may similarly reveal how people view abortion and the associated complexity of belief systems at a population-level scale. Notably, the inescapable role of social media in the public lexicon has evolved over time into an outlet for community building and information dissemination that can connect users over shared interests disregarding location [14]. For example, the Pew Research Center contends that more than three-quarters of the US adult population regularly use at least 1 social media platform [15], and half of all the users have actively maintained at least 1 account for more than a decade. Because social media data are part of the public domain, longitudinal tracking of such data can represent an open-access running diary of thoughts, perspectives, and affective indicators—particularly for issues deemed controversial or contentious, including COVID-19 vaccination status, marriage equality, transgender sports bans, and abortion [16,17]. Furthermore, social media data are also global, implying that shared languages, regardless of geographic constraint, can contribute to discourses about abortion and associated beliefs therein.

Research has documented that people use social media to share their opinions and views and engage in debates on various topics, as well as to seek help and information and solicit personal advice that pertains to their situation or to something they are going through in life [18-20]. These web-based interactions vary widely across social media platforms and topics but may include discussions about substance use disorders [21], mental health [22], sexual assault [23], and managing HIV treatment [24], among a wide range of other topics. Furthermore, some more limited research has explored social media users’ engagement and interactions as part of sharing personal experiences, soliciting help, and requesting information pertaining to abortion. This research has focused particularly on assessing how social media users rely on each other to discuss cost-related barriers to abortion care [25], to discuss decision-making processes regarding abortion methods [26], and to seek support to make abortion decisions when they may lack familial and medical support otherwise [27].

Reddit (Reddit, Inc) is a social networking website, which is defined by its structure that allows users to subscribe to forums on diverse topics, both controversial and noncontroversial. Their approach to topic discussion is distinct from other social media platforms in that users can opt into conversations with variably different foci depending on needs and interests. For example, previous research has demonstrated that Reddit can serve as a social connection metric, information-sharing tool, and outreach resource [28] for controversial or contentious social topics, including sexual assault [29], abortion [30], and addiction and recovery [21]. For most, Reddit forums are a source of information on these topics. However, many of these same topics, particularly those with political contexts, can also be discussed on different Reddit forums in more social commentary or debate-style perspectives. Abortion is one example of a contentious social topic with ranging subreddits pertaining to different aspects of abortion, including as a social connection and information-sharing tool and debate platform.

Analyzing different facets of the same topic through various subreddits could yield nuanced aspects regarding crucial health topics unique from other quantitative and qualitative abortion research. Notably, as of December 2022, Reddit was the 20th most accessed website globally (sixth in the United States), and 50% of all Reddit users reside in the United States, with Canada, Australia, and the United Kingdom comprising approximately 20% of the total Reddit users. Reddit data can principally serve as a window into views on abortion in the United States; however, because not all English language data originate in the United States, it is also possible to observe abortion attitude complexity in a more Westernized, but global context or global reactions to news related to abortion in the United States.

### Objectives

Advances in computational data mining have made it feasible to extract, analyze, and interpret these data en masse. This study used natural language processing (NLP) and data mining methods to identify and visualize latent themes across 2 distinct subreddits specific to abortion: r/Abortion and r/AbortionDebate. As a comparative study, we aimed to compare the semantic and content differences across these subreddits to gain a comprehensive social media portrait of abortion dialogue on Reddit. This study was guided by three research questions:

1. What themes emerge in a corpus of Reddit posts in r/Abortion, the largest subreddit dedicated to abortion social support and outreach?
2. What themes emerge in a corpus of Reddit posts in r/AbortionDebate?
3. What do similarities and differences by subreddits implicate regarding social media–derived beliefs and ideologies on abortion?

## Methods

### Data

Data for this study were collected over 5 months (ie, from January 2020 to May 2022) from the social networking website Reddit. Reddit represents an open network of communities where users can engage and connect with others over shared interests, hobbies, or personal experiences. Unlike other popular social media websites used for computational analyses, including X (X Corp, formerly known as Twitter), Reddit is unique in that users can create specific channels to form communities with other interested parties on diverse issues or topics. These channels, otherwise known as subreddits, comprise people with shared identities who find, subscribe to, and post within these channels. For instance, people interested in gaming can join the r/Gaming subreddit and people with depression can join the r/Depression subreddit.

We leveraged the PRAW (Python Reddit Application programming interface Wrapper) [31], a third-party application programming interface (API), to collect data for this study and specifically to isolate and download content posted into subreddits in English germane to abortion—we queried the API to allocate similar subreddits also spanning abortion-related topics. This query returned 1 additional subreddit: r/AbortionDebate. Given observable differences in framing (ie, people’s abortion experiences vs debates about abortion), we included this subreddit in our study as an additional but mutually distinct unit of analysis; that is, we collected and stored data for r/Abortion and r/AbortionDebate as separate corpora intended for separate analyses. All data collected for this study were in English, which we selected for 2 reasons: first, >70% of all Reddit users originated from English-speaking countries, and second, at the time of data collection, Reddit posts originating in languages other than English were insufficient for analysis. In Spanish, for example, r/Aborto contained only 5 members, with no activity since 2019; similarly, we observed <50 Spanish-language posts in either r/Abortion or r/AbortionDebate.

Once we identified our subreddits of interest, we queried the API to collect new posts and top posts from the r/Abortion and r/AbortionDebate subreddits. After filtering our data for duplicates and accounting for API data scraping limits, our final sample size comprised 4966 posts, divided into 2 corpora: 56.68% (2815/4966) of r/AbortionDebate posts and 43.31% (2151/4966) of r/Abortion posts.

### Analyses

We aimed to use NLP to identify salient categories in the r/Abortion and r/AbortionDebate subreddits. In numerous studies, latent Dirichlet allocation (LDA) topic models have been predominantly used for this purpose. LDA is a well-regarded unsupervised probabilistic model that evaluates word co-occurrence patterns using an iterative Gibbs sampling method [32]. Although LDA is often considered the gold standard within many academic and professional communities, advancements in NLP, artificial intelligence, and neural networks have introduced innovative topic modeling methods that can more closely approximate the potential meaning in these categories [33].

For this study, we applied one such advancement, the Bidirectional Encoder Representations from Transformers (BERT) topic modeling tool, BERTopic. BERTopic is an NLP topic modeling approach used to identify latent themes or topics within a collection of interrelated documents [34]. Unlike LDA, which uses probabilistic modeling to identify latent topics, BERTopic leverages pretrained embeddings from one of many transformer models, a type of neural network architecture in which an input sequence is compared against large-scale language models to calculate embeddings [35]. Embeddings are used to convert unstructured data, including words and sentences, into fixed-length continuous vectors. These vectors enable mathematical operations to capture semantic meanings, relationships, and other properties related to natural human language.

The vectors calculated using this approach tend to be highly dimensional and difficult to interpret. To reduce dimensionality while maintaining the integrity of our data, we applied a principal component analysis, which is commonly applied in NLP approaches for general dimensionality reduction purposes [36]. This analysis allowed us to extract and more easily interpret a range of possible clusters or topics in both the r/Abortion and r/AbortionDebate subreddit data. Once we reduced the dimensionality of our vectors, we applied a Hierarchical Density-Based Spatial Clustering of Applications with Noise to identify latent clusters or topics [37], CountVectorizer to tokenize each topic, and class term frequency–inverse document frequency to extract topic words for each cluster [38].

Furthermore, to gauge the emotional tone or mood represented in each post from the studied corpora, we applied a Valence Aware Dictionary and Sentiment Reasoner (VADER), a rule-based sentiment analysis tool [39], and Text2Emotion, a rule-based emotion analysis tool [40]. VADER sentiment analysis is an algorithm and analysis that examines the polarity of words within each social media post. Posts are fed through a lexicon or web-based dictionary, which is precoded with values for all positive and negative words in the English language. When posts are run through the VADER lexicon, they receive a composite score. Negative VADER values denote lower affect (ie, −0.99 to −0.01), and positive values denote higher affect (ie, 0.01 to 0.99). Although an older tool, VADER is commonly used to assess content affect and emotional affect. In contrast, the Text2Emotion tool for emotion analysis scans each entry for key phrases and terms denoting one of four base emotions: (1) happy, (2) surprise, (3) fear, and (4) sadness. Collectively, these 2 tools can identify potential tonal differences in each post, again implicating the different uses of each subreddit included in the analysis. Both tools have been applied extensively in computational public health studies owing to their ease of access, replicability, and numerous validation studies [16,21,41,42].

### Procedure

**Figure 1:**
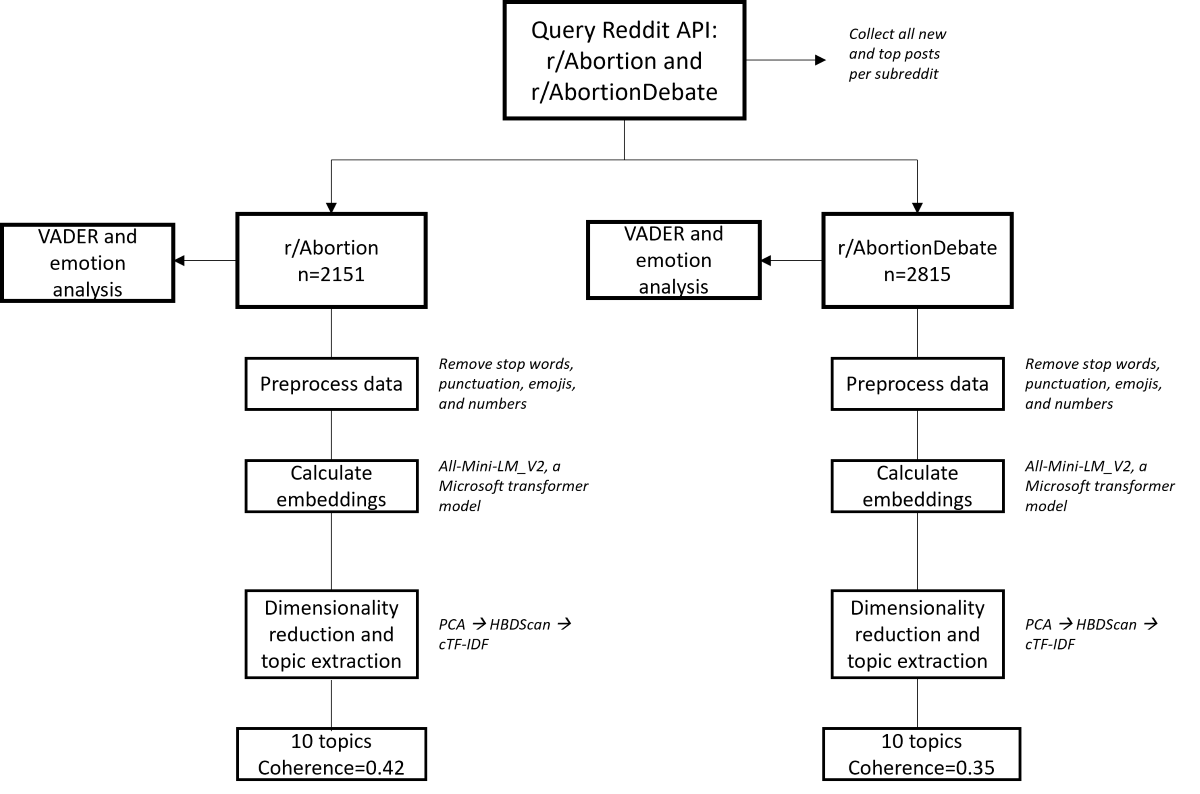
Workflow depicting the study’s methodological pipeline. API: application programming interface; cTF-IDF: class term frequency–inverse document frequency; HBDSCAN: Hierarchical Density-Based Spatial Clustering of Applications with Noise; PCA: principal component analysis; VADER: Valence Aware Dictionary and Sentiment Reasoner.

Our workflow is depicted in Figure 1. First, we queried the Reddit API to archive top and new posts from the r/Abortion and r/AbortionDebate subreddits. Data collected from the r/Abortion and r/AbortionDebate subreddits were saved as separate data files. After removing duplicate and non-English posts in either data file, we applied standard preprocessing steps to remove parts of speech that would detract from the clarity of our models, including articles, prepositions, punctuation, abbreviations, and numbers [43]. Once the data were cleaned, we tokenized our data at the sentence level before calculating embeddings. Once the data were preprocessed and tokenized, we proceeded with our BERTopic pipeline. First, to calculate embeddings in our data we applied all-MiniLM-L6_v2 [44], a transformer-based model developed by Microsoft Corp. This model is designed to be a smaller and more efficient transformer model than larger models, including a generative pretrained transformer or T5, which may make it more appropriate for smaller data sets; however, more research is needed to confirm this notion. Once we calculated embeddings for all sentences in each corpus, we applied a principal component analysis to reduce dimensionality in our data, retaining 5 components. We then ran an iterative topic model ranging from 10 to 80 topics and calculated coherence scores [45] to identify an optimal number of topics, retaining a topic solution with the highest coherence score. For both r/Abortion and r/AbortionDebate, the optimal solution was 10 topics, yielding a respective coherence score of 0.42 and 0.35, which indicates a marginal fit. After we extracted key terms per topic, we applied a sorting function to examine key terms in each entry. Each entry was then classified into one of 10 possible topics in either corpus. We lastly performed a VADER sentiment analysis and Text2Emotion emotion analysis for each entry in both corpora.

### Ethical Considerations

This study involved a secondary analysis of deidentified and anonymized Reddit posts collected between January and May 2022. As this was an observational study with no contact between human subjects and no possible way to trace posts to any individual author, this study was exempt from Institutional Review Board review.

## Results

Our study applied computational tools to collect and analyze subreddits specific to abortion. We aimed to examine how abortion was discussed on the social media platform Reddit, both as an information-sharing tool and as a platform for debating worldviews.

### What Themes Emerged in a Corpus of Reddit Posts About r/Abortion, the Largest Subreddit Dedicated to Abortion Social Support and Outreach?

Our coherence score analysis indicated a 10-topic solution for the r/Abortion subreddit. Table 1 outlines each topic by keywords, the number of sentences belonging to each topic, and the percentage of each topic relative to the larger corpus. Names for each topic were derived by reviewing a small excerpt of Reddit data that were sorted into one of 10 topics by a sorting function using keywords.

The r/Abortion subreddit analysis revealed numerous ways in which abortion was discussed in a social support context. The most prominent topic of our study, *topic 1: sharing support*, comprised the bulk of the conversation with >18% total representation. Social support was commonly manifested by people sharing their own experiences with abortion or by friends and family members who may have experienced abortion. This was further evident by multiple topics containing information-sharing content: *abortion experience* (topics 3, 7, and 10). Beyond social support, several of our topics also appeared to discuss abortion in a neutral and educational information-sharing context: *general abortion* (topic 5) and *general pregnancy* (topic 8).

Further review of the topics added context to our findings. Table 2 provides a summary of each topic and the key excerpts that denote additional meaning. As shown in Table 2, there was little content indicative of debate or questioning one’s position on abortion. Instead, we observed personal experience sharing, including narrative accounts of one’s experience with abortion generally, miscarriage, and medication abortion specifically.

**Table 1:** Topic names and key terms pertaining to r/Abortion subreddit posts (n=2151).

Index	Name	Key terms	Sentences (n=42,732), n (%)
1	Sharing support	want, wanted, told, didnt, say, asked, said, talk, okay, dont	7970 (18.65)
2	Postabortion emotion	feel, sadness, feeling, depressed, regret, depression, emotionally, guilt, grieving, emotional	4502 (10.54)
3	Abortion experience	slept, pad, bed, hour, rest, test, waited, room, home, minute	4361 (10.21)
4	Social support	therapy, supportive, support, struggling, advice, experience, situation, care, family, talked	4215 (9.86)
5	General abortion	abortion, pregnancy, aborted, miscarriage, fetus, pregnant, abort, parenthood, birth, prochoice	4093 (9.58)
6	Clinic experience	appointment, clinic, pill, prescription, sedation, patient, consultation, doctor, iud, obgyn	3770 (8.82)
7	Abortion experience	anesthesia, sedation, anxiety, painkiller, pain, ibuprofen, relief, anxious, painful, uncomfortable	3584 (8.39)
8	General pregnancy	pregnancy, pregnant, gestational, miscarriage, condom, positive, placenta, fetus, parenthood, pill	3441 (8.05)
9	Abortion alternatives	pregnant, pregnancy, adoption, child, parent, selfish, baby, kid, mother, marriage	3433 (8.03)
10	Abortion experience	cramp, crampy, cramping, bleeding, bleed, clot, ibuprofen, spotting, period, nausea	3363 (7.87)

**Table 2:** r/Abortion subreddit summaries with example excerpts.

Topic name	Summary	Excerpts
Sharing support	This cluster contained diverse content pertaining to lending and sharing abortion-related support.	“I just wanted to say that I am thinking of you during this time” “Do not let the extremist propaganda get to your head.” “This subreddit (r/Abortion) is a (mostly minus the trolls) community.”
Postabortion emotion	This cluster contained content pertaining to sharing emotions associated with abortion decision-making and postabortion procedures.	”I know I made the right decision; but I will always grieve you.” “I just want to vent my frustrations and emotions here.” “I still have my moments of sadness but overall, I would make the same decisions again if I needed to.”
Abortion pre- and postexperience	This cluster contained content pertaining to pre- and postabortion experiences, including decision-making and outcomes.	“When I waited I could not believe the amount of protestors outside.” “I’m panicking in my dreams and then I wake up and realize I still have six days to go.” “When she (my girlfriend) found out two weeks ago, she was around 11 weeks along”
Social support	This cluster contained content pertaining to social support related to abortion care.	“I would like to offer a free ride to such locations (counselors) for anyone in need.” “I also want to thank the volunteer escorts who helped me to and from my car.” “If you are a volunteer escort, your actions and help are so important.”
General abortion	This cluster contained general abortion content, usually in a supportive context.	“Women who seek abortions are often vilified whereas women who give birth are celebrated.” “Abortion is health care, abortion is birth control.” “Since my abortion I have graduated high school, graduated an associates program, a bachelors program, and am in grad school.”
Clinic experience	This cluster contained content pertaining to individual experiences at abortion care and general health care facilities.	“My ultrasound was this morning and everyone at the clinic was rude.” “I had a good experience at the clinic, the staff were very considerate and kind.” “At the location I went to they had setup a fake clinic across the street and bought the house next door.”
Abortion experience	This cluster contained content pertaining to pre- and postabortion experiences, including anticipating an abortion procedure.	“However the protestors yelling at you while you walk into the clinic is ridiculous.” “I am also terrified about the pain- they do not offer anesthesia, just pain meds.” “I ended up hemorrhaging pretty badly and had to be taken to the hospital via ambulance.”
General pregnancy	This cluster contained content pertaining to general pregnancy discussions, including accidental pregnancy and miscarriage.	“10 weeks into the pregnancy, I miscarried.” “15 years later and I intentionally became pregnancy.” “Six months later, I intentionally stick my foot through the pregnancy door again.”
Abortion alternatives	This cluster contained diverse content pertaining to abortion alternatives.	“I considered adoption, but abortion was right for me at that time.” “Forced births are not the answer.” “Can anyone logically think of true alternatives to abortion?”
Abortion experience	This cluster contained content pertaining to abortion experiences through a medication abortion lens.	“Did you experience different side effects, was the bleeding/clots different?” “My paper says tramadol every 2 hours, ibuprofen every 6 hours, and Tylenol every 4.” “It took me about 16 hours after the misoprostol to start bleeding and it was extremely heavy and clotty.”

Perhaps one of the most recurring patterns in our data was frank discussions about postabortion feelings in a clinical setting, (“I felt so nauseous in that waiting room, I was not sure I could go through with it”), a postabortion setting (“It took me a few days to finally feel like myself again post-abortion”), or a medication abortion context (“The mifepristone caused some pretty intense clotting after I took the pill”). The medication abortion narratives were sometimes framed as someone explaining their decision (“I chose the pill because where I live you cannot have someone with you when getting an abortion due to COVID-19”).

### What Themes Emerged in a Corpus of Reddit Posts in the r/AbortionDebate Subreddit?

Our coherence score analysis indicated a 10-topic solution for the r/AbortionDebate subreddit. Table 3 outlines each topic by keywords, the number of sentences belonging to each topic, and the percentage of each topic relative to the larger corpus. Names for each topic were derived by reviewing a small excerpt of Reddit data that was sorted into one of 10 topics by a sorting function based on keywords.

Unlike the r/Abortion subreddit, which we determined seemed to be used in a social support and information-sharing context, the r/AbortionDebate subreddit comprised conversations dedicated to critically assessing abortion from legal, moral, and ethical perspectives. The topic with the greatest representation was *topic 1: Reddit forum rules and regulations*. In topic 1, we observed several posts directly from moderators explicitly warning against outright attacks, misinformation, and vitriol targeted at people with opposing views on abortion; this topic was absent completely in the r/Abortion topic model. The second most prominent topic, *topic 2: abortion morality*, was centered on debating abortion from a moral perspective. The topic with the smallest representation was topic 5, pertaining to *general pregnancy*. At face value, we did not observe a great overlap in topic content in the r/AbortionDebate subreddit compared with the r/Abortion subreddit. However, we reviewed additional excerpts to ascribe a deeper meaning to these topics to examine precisely how abortion debates manifested on these forums.

Table 4 outlines additional information about each topic, including a summary and key excerpts that implicate deeper meaning. This additional analysis allowed us to examine more precise moral, legal, and ethical arguments pertaining to people’s expressed views on abortion.

**Table 3:** Topic names and key terms pertaining to the r/AbortionDebate subreddit posts (n=2815).

Index	Topic name	Key terms	Sentences (n=30,031), n (%)
1	Forum rules	post, rule, problem, posted, following, response, sorry, line, mod, issue	5847 (19.47)
2	Abortion morality	morality, debating, discussion, arguing, debate, argue, reasoning, argument, premise	3253 (10.83)
3	Abortion legality	law, enforcement, violence, consent, crime, autonomy, consenting, freedom, murder, bodily	3240 (10.79)
4	Personhood	abortion, parenthood, fetus, unborn, pregnancy, aborted, contraception, maternal, abort	2839 (9.45)
5	General pregnancy	prenatal, pregnancy, childbirth, fetus, maternal, pregnant, fetal, gestational, placenta	2754 (9.17)
6	Abortion debate	abortion, debate, fetus, prolife, argument, arguing, morality, advocate, prolifers	2700 (8.99)
7	Humanity and life	human, humanity, biological, personhood, sentient, moral, consciousness, concept, brain	2573 (8.57)
8	Euthanasia	euthanasia, killing, death, dying, murder, lethal, suicide, kill, die, killed	2325 (7.74)
9	Abortion alternatives	pregnancy, parenthood, contraception, abstinence, pregnant, contraceptive, fetus, unborn	2320 (7.72)
10	General pregnancy	fetus, foetus, unborn, fetal, conception, womb, embryo, pregnancy, childbirth, pregnant	2185 (7.27)

**Table 4:** r/AbortionDebate subreddit summaries with example excerpts.

Topic name	Summary	Excerpts
Forum rules	This cluster contained content from moderators reminding posters to mind rules and regulations.	“As a courtesy, be respectful to others.” “Rules on this forum are non-negotiable.” “We will remove content that does not ascribe to our rules.”
Abortion morality	This cluster contained diverse content on abortion morality, including acceptability and permissibility.	“Title is kind of a joke but the question is serious: I’m wondering what you all think are the moral arguments for your ‘side’ of the debate.” “Do you have a clear point where you draw the line?” “Real-time discussion will hopefully lead to better overall conversation about right versus wrong.”
Abortion legality	This cluster contained content on abortion laws, legality, and future legal maneuvers to restrict abortion.	“Creating a law that only affects 50% of the population is discrimination no matter how you look at it.” “Similar measures have in the past been filed by state Rep. Tony Tinderholt, R-Arlington, who received death threats and was placed under the protection of the Texas Department of Public Safety after he introduced the bill in 2017.” “And the Texas law is going to be copied across the country.”
Personhood	This cluster contained discussions about personhood and pregnancy.	“In early stages, can we really say a zygote is a person?” “These laws are not restrictive to the fetus, if you think about it?” “But really, at what point is abortion the murder of a person?”
General pregnancy	This cluster contained diverse content pertaining to pregnancy, including personal experiences and general facts.	“The average newborn head is 11.1 cm in diameter.” “The first week after conception is pretty much taken up with travel plans and implantation.” “Within 8 years teen pregnancy dropped 54%.”
Abortion debate	This cluster contained content pertaining to general abortion debates and dialogues.	“I agree that abortion is murder but it is not okay to harass women outside planned parenthood!” “I disagree that abortion is murder, but there is a limit.” “It’s important to have an honest conversation about abortion to understand all sides.”
Humanity and life	This cluster contained content pertaining to humanity and life with some insights into religion and spirituality.	“There is no sin in keeping your religious beliefs to yourself while allowing others their right to choose.” “I know most of you right-wing Christians work hard for a living.” “To me an essential part of being a human being is that that human being is something/someone I can hold in my hands.”
Euthanasia	This cluster contained content that used euthanasia as a talking point when discussing abortion beliefs.	“Euthanasia/assistant suicide the same, the military most people go into that with the idea that they might have to kill a person, so I think that check out.” “I meet and argued with a lot of pro-lifers, I brought up both the death penalty and euthanasia!” “Not once can I recalled a time where a pro-lifer have use it’s a separate dna to attack the death penalty or euthanasia.”
Abortion alternatives	This cluster discussed content that implicated the complexity of alternatives to abortion, including adoption and the foster system.	“The foster care and adoption systems are already overfilled and refusing to accept anyone else.” “You could just put it up for adoption. That’d be fine if the problem was just parenthood, but it’s not.” “Which when the woman don’t want the offspring for various reasons (personal cost of altruism), they decide the personal cost of the act of parental altruism is too high.”
General pregnancy	This cluster contained content that used pregnancy as a discussion point about abortion rights, morality, and legality.	“Unborn humans literally live inside of someone else.” “On a post like a week ago I read that some PC folks are frustrated with the test tube of embryos vs infant in a burning building argument.” “If you are pro-choice, do you also adopt the anti-natalist perspective?”

For example, we observed that the abortion morality topic typically contained content related to drawing lines about abortion permissibility (“Where do [people] draw the line between acceptable and not acceptable”). This style of discussion was mirrored in conversations about fetal personhood (“Who here honestly believes a zygote is a person with rights?”) and the role spirituality plays in moral arguments about abortion (“But what do Catholics really think on this issue?”). Discussions and arguments about abortion morality were notably similar to the content in *topic 3: abortion legality*. Content on this topic typically discussed new abortion-related laws, the merits of those laws, and opinions about their relative effectiveness (“Texas passed a very restrictive law and it will serve as a benchmark for other states, watch”). Importantly, and across topics, we observed that people declared their abortion views (“I am pro-choice and I will always be”) and, in some cases, discussed how their abortion views evolved over time (“I am pro-life, but we should be discussing the merits of abortion as a life-saving tool here”). Here, we observed more opinions than the outright support articulated in the r/Abortion subreddit.

### What Do Similarities and Differences by Subreddit Implicate About Social Media–Derived Abortion Beliefs and Ideologies?

**Figure 2:**
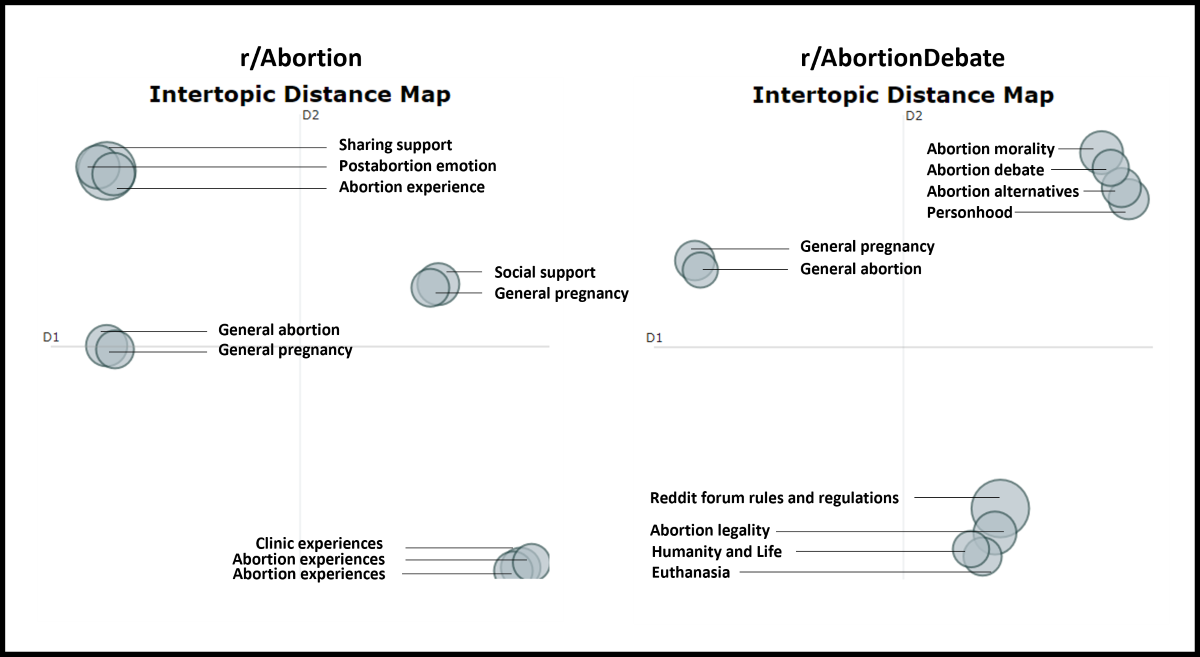
Intertopic distance map indicating relative similarity and difference per topic per corpus. D1 and D2 refer to the 2 retained components for visualization purposes and are not meant to be interpreted.

Figure 2 visually represents data from each subreddit, where dense, overlapping clusters signify similar topics (or higher collinearity) and nonoverlapping circles indicate dissimilar topics (or lower collinearity).

In both the r/Abortion and r/AbortionDebate subreddits, the intertopic distance maps depict mutual exclusivity in general abortion and pregnancy topics, distinguished by basic sharing of language and specific information related to pregnancy and abortion (“Sometimes a pregnancy can end without warning or reason”; “abortion is a women’s health issue”). Beyond these statements, however, other conversations exhibit a richer and more nuanced discourse about abortion, overlapping between topics and offering deeper insights into an individual’s worldview on abortion, and portraying how various co-occurring factors influence one’s beliefs and worldviews (“Laws are one thing but have you considered the humanistic side of it all?”).

We used VADER and Text2Emotion tools to discern affective differences between r/Abortion and r/AbortionDebate subreddits. The r/Abortion subreddit displayed a compound VADER score of 0.10, reflecting overall neutral content, whereas the r/AbortionDebate subreddit displayed a score of −0.06, denoting neutral to slightly negative content. The emotion analysis findings for the r/Abortion subreddit were as follows: *happy* (mean 0.06, SD 0.19)*, angry* (mean 0.20, SD 0.31)*, surprise* (mean 0.12, SD 0.26)*, sad* (mean 0.20, SD 0.31)*,* and *fear* (mean 0.36, SD 0.39)*.* The emotion analysis findings for r/AbortionDebate subreddit were as follows: *happy* (mean 0.12, SD 0.27), *angry* (mean 0.05, SD 0.18), *surprise* (mean 0.11, SD 0.25), *sad* (mean 0.22, SD 0.35), *fear* (mean 0.28, SD 0.35).

Furthermore, the Text2Emotion variable *fear* was prominent in r/Abortion, whereas *happy* was slightly more elevated in r/AbortionDebate. These observed differences are likely attributed to the differing nature and scope of the subreddits. For instance, the manifestation of *fear* may be more related to personal abortion narratives in r/Abortion, whereas happiness may arise from occasional friendly exchanges of views in r/AbortionDebate.

Despite their different foci, both subreddits contain myriad conversation topics, allowing for civil and enlightening discussions on evolving abortion views and ideologies. The discourse in these forums sometimes hints at the evolution of individual ideologies with time, reflecting the dynamic nature of personal beliefs and the influences shaping them (adapted excerpt: “I guess I just don’t know my views”; excerpt: “My opinion changed over time, growing up in a Christian household I was always against abortion...until I needed one myself”)*.* This phenomenon underscores the essential role of such platforms in fostering understanding and dialogue on the multifaceted issue of abortion.

## Discussion

Our study leveraged Reddit data as a novel, big data form of qualitative data to examine abortion discourse on r/Abortion and r/AbortionDebate subreddits. We observed several important themes, including evidence of complexity in abortion-related social media posts, which warrant further discussion.

### The r/Abortion Subreddit as an Information-Seeking or Information-Sharing Platform for People With Questions About Their Abortion Experiences

Within the r/Abortion subreddit, we noticed posters using this platform to discuss abortion in diverse, sometimes overlapping contexts. However, each topic emerging from r/Abortion typically involved a degree of information sharing, whether through the provision of available resources or sharing personal narratives and experiences with abortion. We primarily observed these types of posts in *topic 1: sharing support*; *topic 2: postabortion emotions*; *topics 3, 7, and 10: abortion experience*; and *topic 5: clinical experience*. The content within these topics typically involved direct sharing of one’s experiences related to abortion or posing highly specific questions about access (eg, excerpt: “Is abortion legal past 6 weeks gestation in Oklahoma?”) and medication abortion (eg, excerpt: “Abortion is legal here; can I get abortion pills by mail?”). Within the medication abortion topic, the content was both informative and supportive, with some posters sharing their experience in solidarity with others facing a similar choice. Notably, we did not observe any critiques against anyone’s abortion narratives; rather, the tone and structure, as also evident in this study’s VADER and emotion analysis, are largely informative and overall supportive of abortion. Given that the rules and guidelines established this subreddit as a place of nonconfrontational discussion, perhaps people advocating for other reproductive choices may have shared their perspectives in other subreddits, such as those related to adoption.

We acknowledge the possible connection between personal tendencies to share intimate information and the continually evolving role of the internet as a medium for social connection and information acquisition [46]. Notably, for the past 3 decades, the internet has become the most influential medium for information-seeking globally. The Pew Research Center indicates that approximately 80% of the adult population in the United States regularly use the internet to acquire general information or understand unfamiliar topics [47]. For example, an individual contemplating an abortion might opt to seek guidance in web-based forums to avoid potential ostracism from friends and family. Similarly, a friend or family member of someone considering an abortion might turn to web-based forums to secure advice or perspectives on assisting their loved one. Discourse on such platforms is crucial, especially when addressing sensitive topics that many may feel uneasy to discuss openly. This emphasizes the significance of the internet as a confidential and reliable resource for information and advice. Importantly, this also supports Reddit as a source of information for people needing abortion-related counseling.

These excerpts, and others in our composite sample, illustrate that social networking websites serve as a potentially crucial source of information for some [48], offering insights and details that may be otherwise unavailable, including local and state resources for abortion. This finding becomes particularly salient in light of the overturning of *Roe v. Wade*, which marked the end of federal protections for abortion until viability [49]. In the wake of this decision, 24 states enacted bans with limited exceptions or additional restrictions on abortion—generally earlier in terms of weeks of gestation than previously occurring under *Roe v. Wade* [50]. For those residing in states where abortion transitioned from being broadly legal to almost entirely illegal, web-based resources may have played a pivotal role during instances of unplanned pregnancy, as observed previously [51]. Further research is imperative to assess the efficacy of Reddit and other social networking sites in offering support and resources on this and other health-related topics. Notably, this subreddit contained little to no expression about personal abortion beliefs and ideologies.

### The r/AbortionDebate Subreddit and Discussions of Abortion Identity and Changing Views Over Time

We did not observe much information or support sharing in the r/AbortionDebate subreddit. Rather, content in this subreddit discussed values and beliefs about abortion across many domains, including ethical, moral, legal, and humanistic. In several circumstances, we observed complex and nuanced abortion perspectives that do not correspond neatly to prochoice or prolife frameworks—2 commonly used but contested abortion identity labels used to outline personal abortion beliefs. For example, as many as half of the topics uncovered by r/AbortionDebate contained contradictory expressions regarding abortion and how the abortion debate was framed. These posts were broadly delineated as those deconstructing or debating prochoice and prolife movements and others explaining how circumstances contributed to moral and ethical shifts in abortion views, for example, in the following excerpts: “I was and will always be pro-choice, but my reaction was absolutely not [to abort a fetus with serious birth defects] even though I knew it was the right answer” and “I was pro-life and never thought I’d need Planned Parenthood until I did. My experience changed my opinion of them, but [I still wish] they didn’t primarily exist to perform abortions.” Here, the emphasis is far less on information or support sharing, rather the purpose is to articulate personal views about abortion and defend them accordingly. These findings align with ongoing abortion attitude research citing complex or nuanced abortion views that do not neatly fit into a singular label [52-54].

In addition to discussing and debating abortion values, we observed more combative content in the r/AbortionDebate subreddit. This is likely by design, namely to parse out people seeking information about abortion versus people looking to debate abortion [55]. Such differences between the r/Abortion and r/AbortionDebate subreddits were particularly evident in our sentiment and emotion analyses. For example, r/AbortionDebate yielded slightly more negative VADER affect scores and decreased emotion analysis scores for *fear*. We attributed more negative VADER scores to the often contentious exchanges among users (excerpt: “All these pro-choicers in here trying to lump as all as anti-women bigots”). We attributed lower *fear* scores to the apparent use of r/AbortionDebate as a forum to discuss abortion views and not for sharing information or narrative accounts about abortion. In other words, negative language was reflected via discourse in the r/AbortionDebate subreddit, as opposed to expressing personal fears or concerns about abortion, which may have surfaced more in the r/Abortion subreddit. In this context, the r/AbortionDebate subreddit may be more useful for mining insights into abortion ideologies, particularly when examining precise factors about abortion, including moral and legal arguments, gestational limits, and others. However, to gain insights into how abortion, as a medical procedure, is communicated from a decision-making perspective, r/Abortion may be more informative.

We identified 2 main implications from the content differences observed in r/Abortion and r/AbortionDebate. First, opting for the right Reddit forum is critically important. Reddit’s structure—where users select forums based on interests or needs—is different from other social networking sites. For people looking for ideally accurate, impartial information about abortion, r/Abortion or similar subreddits are suitable. Meanwhile, r/AbortionDebate is better for those wanting to discuss and ponder the ethical aspects of abortion. However, this choice is dependent on knowing how Reddit works. We project that a significant proportion of people may join the wrong forum and get exposed to unintended outcomes and viewpoints owing to a lack of preexisting knowledge about Reddit and its operations. Second, our observations support the idea that Reddit’s higher moderation levels make it a valuable tool for social science research. Historically, Reddit has carried the reputation of fostering trolls and hate speech. However, for health content, subreddits tend to be more effectively moderated by content experts. As evidenced in our data, both subreddits seemed relatively free from hate speech and trolling because of this moderation, which is unique to Reddit compared with other social media platforms. Therefore, Reddit remains a fairly reliable platform for both users and researchers, especially in the wake of recent changes in APIs and data access on other platforms, including X (X Corp, formerly known as Twitter).

### Social Media as a Resource and Triangulation Tool to Support Ongoing Quantitative and Qualitative Research on Abortion

Our findings, particularly those critiquing abortion identity labels or people explaining their contextual abortion beliefs, support extant research demonstrating that people’s attitudes toward abortion are complex. Notably, this larger body of research argues that abortion attitudes are not unidimensional or polar but rather vary along legal, moral, social, and other similar domains [2,3,56,57]. This work is composed of both quantitative (surveys) and qualitative (interviews) data collections, which collectively yield deep insights into social attitude formation in the United States and how beliefs vary based on context and other dimensions. Consistent with these studies, our results support the notion that abortion attitudes and abortion decision-making are not unidimensional but involve multiple co-occurring considerations.

The novel nature of social media as data adds additional validity to previous abortion attitude research. This is particularly salient regarding how our findings triangulate or corroborate previous research on abortion attitude complexity. Notably, by mining Reddit abortion forums, we observed at least two principal uses of these forums: (1) as a space to share narratives and resources about abortion and (2) as a dedicated channel to debate abortion views. For many, Reddit forums could be a place where some people feel comfortable sharing or debating abortion views, although we acknowledge that more research on this area is needed. Furthermore, Reddit offers a somewhat anonymous space where people can gather the information they need about abortion or inform their perspectives on abortion. These shared Reddit perspectives, which are generally top of the mind, spontaneous, and unprompted [58], may provide a window into collective abortion beliefs that support or refute previous findings from other conventional forms of data collection. Similar uses of social media data, namely to corroborate findings on social issues, including gun control [59], marriage equality [60], and vaccination mandates [61], have been similarly leveraged. Therefore, we argue that social media can be a valuable source of data to help elucidate people’s opinions on relevant social issues.

Furthermore, we argue that national surveys, strategic qualitative interviews, and mass social media scrapes as data sources yield specific outcomes that, when combined, provide a robust and comprehensive portrait of social issues. Survey data, which are strengthened when participants are identified via probability-based sampling protocols [62], reveal nationwide associations between demographic variables and other variables of interest. Qualitative data can reveal insights into highly specific research questions, for example, whether changing auxiliary verbs leads to diverging responses about abortion beliefs [63]. Social media data scrapes can offer population-level insights that support or contradict findings from previous studies at the population-level scope and scale [41]. Our Reddit data support previous findings from surveys and qualitative research, demonstrating how social media data can serve as a triangulation tool. We contend that further strategic applications of social media mining with traditional quantitative and qualitative research can provide highly accurate portrayals of social views in the United States.

### Limitations and Future Research

This study has several limitations that we hope to address in future research. First, although Reddit posts can be construed as qualitative data, we did not perform a formal qualitative analysis using these data. Owing to the scope of this study, we instead leveraged NLP algorithms to categorize and visualize all data simultaneously. In the future, researchers could perform detailed qualitative inquiries with these data, which can occur with the entire data set or among one or several clusters depending on the scope and research questions. Second, our study was limited to exploratory analyses. Although more refined algorithms could more effectively annotate and classify our data, we believed that these approaches would better serve as a follow-up to our exploratory approach to mining Reddit data. Future studies should consider using our data for more refined machine learning–driven or artificial intelligence-driven tasks. Finally, our study was limited by its relatively small timeframe (5 months). It is likely that collecting data for an even longer period may have yielded more nuanced findings.

### Conclusions

With the decision in *Dobbs v. Jackson Women’s Health Organization* overturning *Roe v. Wade*, there is renewed attention to abortion as a contentious political and social issue. Despite abortion being an exceedingly complex topic, political debate and discussions about abortion are generally framed dichotomously as a support or opposition, or prolife or prochoice issue. However, extensive research indicates that public opinion about abortion does not ascribe neatly to that dichotomy and that circumstances beyond a person’s control may lead to shifts in views of abortion over time. Our research corroborates such findings that detail the myriad ways in which abortion attitudes are complex and contextual, beyond simple information-seeking. Furthermore, our findings provide evidence that social media data can be a helpful triangulation tool for public opinion survey research.

## Abbreviations

API: application programming interface
BERT: Bidirectional Encoder Representations from Transformers
LDA: latent Dirichlet allocation
NLP: natural language processing
PRAW: Python Reddit application programming interface Wrapper
VADER: Valence Aware Dictionary and Sentiment Reasoner

## Data Availability

The data are currently stored in a secure GitHub repository and are available for further analysis upon request.

## References

[1] Jozkowski KN, Crawford BL, Hunt ME (2018). Complexity in attitudes toward abortion access: results from two studies. Sex Res Soc Policy.

[2] Jozkowski KN, Crawford BL, Turner RC, Lo WJ (2019). Knowledge and sentiments of Roe v. Wade in the wake of justice Kavanaugh’s nomination to the U.S. Supreme Court. Sex Res Soc Policy.

[3] Jozkowski KN, Crawford BL, Willis M (2020). Abortion complexity scores from 1972 to 2018: a cross-sectional time-series analysis using data from the general social survey. Sex Res Soc Policy.

[4] Maier JM, Jozkowski KN, Valdez D, Crawford BL, Turner RC, Lo WJ (2021). Applicability of a salient belief elicitation to measure abortion beliefs. Am J Health Behav.

[5] Hans JD, Kimberly C (2014). Abortion attitudes in context: a multidimensional vignette approach. Soc Sci Res.

[6] Crawford BL, LaRoche KJ, Jozkowski KN (2022). Examining abortion attitudes in the context of gestational age. Soc Sci Q.

[7] Smith TW (2007). An evaluation of Spanish questions on the 2006 general social survey. NORC/University of Chicago.

[8] Bowman K, Goldstein S (2021). Attitudes about abortion: a comprehensive review of polls from the 1970s to today. American Enterprise Institute.

[9] Doherty D (2022). What can conjoint experiments tell us about Americans’ abortion attitudes?. Am Politics Res.

[10] Jelen TG, Wilcox C (2016). Causes and consequences of public attitudes toward abortion: a review and research agenda. Polit Res Q.

[11] Buyuker BE, LaRoche KJ, Bueno X, Jozkowski KN, Crawford BL, Turner RC, Lo WJ (2023). A mixed-methods approach to understanding the disconnection between perceptions of abortion acceptability and support for Roe v. Wade among US adults. J Health Polit Policy Law.

[12] Friedersdorf C (2021). There are more than two sides to the abortion debate. The Atlantic.

[13] Adamo C, Carpenter J (2023). Sentiment and the belief in fake news during the 2020 presidential primaries. Oxf Open Econ.

[14] Milakovich ME, Wise JM (2019). Internet technology as a global connector. Digital Learning.

[15] Perrin A (2015). Social media usage: 2005-2015. Pew Research Center.

[16] Bathina KC, Ten Thij M, Valdez D, Rutter LA, Bollen J (2021). Declining well-being during the COVID-19 pandemic reveals US social inequities. PLoS One.

[17] Zafarani R, Abbasi MA, Liu H (2014). Social Media Mining: An Introduction.

[18] Jacques L, Valley T, Zhao S, Lands M, Rivera N, Higgins JA (2023). "I'm going to be forced to have a baby": a study of COVID-19 abortion experiences on Reddit. Perspect Sex Reprod Health.

[19] Priya S, Sequeira R, Chandra J, Dandapat SK (2019). Where should one get news updates: Twitter or Reddit. Online Soc Netw Media.

[20] Ong E, Davis L, Sanchez A, Stohl HE, Nelson AL, Robinson N (2022). A review of women’s unanswered questions following miscarriage on different social media platforms [A207]. Obstet Gynecol.

[21] Valdez D, Patterson MS (2022). Computational analyses identify addiction help-seeking behaviors on the social networking website Reddit: insights into online social interactions and addiction support communities. PLOS Digit Health.

[22] Sit M, Elliott SA, Wright KS, Scott SD, Hartling L (2022). Youth mental health help-seeking information needs and experiences: a thematic analysis of Reddit posts. Youth Soc.

[23] Abavi R, Branston A, Mason R, Du Mont J (2020). An exploration of sexual assault survivors' discourse online on help-seeking. Violence Vict.

[24] Ayers JW, Zhu Z, Harrigian K, Wightman GP, Dredze M, Strathdee SA, Smith DM (2023). Managing HIV during the COVID-19 pandemic: a study of help-seeking behaviors on a social media forum. AIDS Behav.

[25] Higgins J, Lands M, Valley T, Carpenter E, Jacques L (2021). Real-time effects of payer restrictions on reproductive healthcare: a qualitative analysis of cost-related barriers and their consequences among U.S. abortion seekers on Reddit. Int J Environ Res Public Health.

[26] Jacques L, Carpenter E, Valley T, Alvarez B, Higgins J (2021). Medication or surgical abortion? An exploratory study of patient decision making on a popular social media platform. Am J Obstet Gynecol.

[27] Richards NK, Masud A, Arocha J (2020). P28 Breaking down abortion barriers: Reddit users’ empowerment in absence of parental and medical support. Contraception.

[28] Sawicki J, Ganzha M, Paprzycki M, Watanobe Y (2023). Reddit CrosspostNet—studying Reddit communities with large-scale Crosspost graph networks. Algorithms.

[29] Lanthier S, Mason R, Logie CH, Myers T, Du Mont J (2023). "Coming out of the closet about sexual assault": intersectional sexual assault stigma and (non) disclosure to formal support providers among survivors using Reddit. Soc Sci Med.

[30] Richards NK, Masud A, Arocha JF (2024). Online abortion empowerment in absence of parental and medical support: a thematic analysis of a reddit community’s contributions to decision-making and access. Research Square. Preprint posted online May 24, 2021.

[31] Madan P (2023). Web scraping Reddit with python: a complete guide with code. GoLogin.

[32] Blei DM, Ng AY, Jordan MI (2003). Latent dirichlet allocation. J Mach Learn Res.

[33] Resnik P, Armstrong W, Claudino L, Nguyen T, Nguyen VA, Boyd-Graber J (2015). Beyond LDA: exploring supervised topic modeling for depression-related language in Twitter. Proceedings of the 2nd Workshop on Computational Linguistics and Clinical Psychology: From Linguistic Signal to Clinical Reality.

[34] Egger R, Yu J (2022). A topic modeling comparison between LDA, NMF, Top2Vec, and BERTopic to demystify Twitter posts. Front Sociol.

[35] Wolf T, Debut L, Sanh V, Chaumond J, Delangue C, Moi A, Cistac P, Rault T, Louf R, Funtowicz M, Davison J, Shleifer S, von PP, Ma C, Jernite Y, Plu J, Xu C, Scao TL, Gugger S, Drame M, Lhoest Q, Rush A (2020). Transformers: state-of-the-art natural language processing. Proceedings of the 2020 Conference on Empirical Methods in Natural Language Processing: System Demonstrations.

[36] Drikvandi R, Lawal O (2020). Sparse principal component analysis for natural language processing. Ann Data Sci.

[37] Stewart  G, Al-Khassaweneh M (2022). An implementation of the HDBSCAN* clustering algorithm. Appl Sci.

[38] Kim SW, Gil JM (2019). Research paper classification systems based on TF-IDF and LDA schemes. Hum Cent Comput Inf Sci.

[39] Hutto C, Gilbert E (2014). VADER: a parsimonious rule-based model for sentiment analysis of social media text. Proc Int AAAI Conf Web Soc Media.

[40] Aslam N, Rustam F, Lee E, Washington PB, Ashraf I (2022). Sentiment analysis and emotion detection on cryptocurrency related tweets using ensemble LSTM-GRU model. IEEE Access.

[41] Valdez D, Ten Thij M, Bathina K, Rutter LA, Bollen J (2020). Social media insights into US mental health during the COVID-19 pandemic: longitudinal analysis of Twitter data. J Med Internet Res.

[42] Adarsh R, Patil A, Rayar S, Veena KM (2019). Comparison of VADER and LSTM for sentiment analysis. Int J Recent Technol Eng.

[43] Nesca M, Katz A, Leung C, Lix L (2022). A scoping review of preprocessing methods for unstructured text data to assess data quality. Int J Popul Data Sci.

[44] Hertling S, Portisch J, Paulheim H (2024). KERMIT -- a transformer-based approach for knowledge graph matching. arXiv. Preprint posted online April 29, 2022.

[45] O’Callaghan D, Greene D, Carthy J, Cunningham P (2015). An analysis of the coherence of descriptors in topic modeling. Expert Syst Appl.

[46] Szymkowiak A, Melović B, Dabić M, Jeganathan K, Kundi GS (2021). Information technology and Gen Z: the role of teachers, the internet, and technology in the education of young people. Technol Soc.

[47] Auxier B, Anderson M (2021). Social media use in 2021. Pew Research Center.

[48] Frey E, Bonfiglioli C, Brunner M, Frawley J (2022). Parents' use of social media as a health information source for their children: a scoping review. Acad Pediatr.

[49] Crawford BL, Simmons MK, Turner RC, Lo WJ, Jozkowski KN (2023). Perceptions of abortion access across the United States prior to the Dobbs v. Jackson Women's Health Organization decision: results from a national survey. Perspect Sex Reprod Health.

[50] Tracking abortion bans across the country. The New York Times.

[51] Reis BY, Brownstein JS (2010). Measuring the impact of health policies using internet search patterns: the case of abortion. BMC Public Health.

[52] Kim T, Steinberg JR (2023). Individual changes in abortion knowledge and attitudes. Soc Sci Med.

[53] Bueno X, Asamoah NA, LaRoche KJ, Dennis B, Crawford BL, Turner RC, Lo WJ, Jozkowski KN (2023). People's perception of changes in their abortion attitudes over the life course: a mixed methods approach. Adv Life Course Res.

[54] Jozkowski KN, Mena-Meléndez L, Crawford BL, Turner RC (2023). Abortion stigma: attitudes toward abortion responsibility, illegal abortion, and perceived punishments of “illegal abortion”. Psychol Women Q.

[55] Shen Q, Rosé CP (2022). A tale of two subreddits: measuring the impacts of quarantines on political engagement on Reddit. Proc IntAAAI Conf Web Soc Media.

[56] Crawford BL, Jozkowski KN, Turner RC, Lo WJ (2021). Examining the relationship between Roe v. Wade knowledge and sentiment across political party and abortion identity. Sex Res Soc Policy.

[57] LaRoche KJ, Jozkowski KN, Crawford BL, Haus KR (2021). Attitudes of US adults toward using telemedicine to prescribe medication abortion during COVID-19: a mixed methods study. Contraception.

[58] Kulkarni V, Kern ML, Stillwell D, Kosinski M, Matz S, Ungar L, Skiena S, Schwartz HA (2018). Latent human traits in the language of social media: an open-vocabulary approach. PLoS One.

[59] Dowler K (2002). Media influence on attitudes toward guns and gun control. Am J Crim Just.

[60] O'Connor C (2017). 'Appeals to nature' in marriage equality debates: a content analysis of newspaper and social media discourse. Br J Soc Psychol.

[61] Chen L, Ling Q, Cao T, Han K (2020). Mislabeled, fragmented, and conspiracy-driven: a content analysis of the social media discourse about the HPV vaccine in China. Asian J Commun.

[62] Catania JA, Dolcini MM, Orellana R, Narayanan V (2015). Nonprobability and probability-based sampling strategies in sexual science. J Sex Res.

[63] Maier JM, Jozkowski KN, Montenegro MS, Willis M, Turner RC, Crawford BL, Lo WJ (2021). Examining auxiliary verbs in a salient belief elicitation. Health Behav Policy Rev.

